# Notes and News

**Published:** 1894-08-18

**Authors:** 


### Notes and News.

Collected and Communicated.

The degree of Doctor of Laws has just been conferred for the first time by the University of Rome on a woman, Signorina Teresina Labriola. She is only eighteen years of age.

A yearly pension of 2,000 florins has been granted to the widow of the late Professor Billroth by the Emperor of Austria.

Specimens of the "plague" microbe were exhibited by Mr. Ernest Hart at the museum in connexion with the recent congress of the British Medical Association at Bristol, supplied to him by Dr. Cantlie, of Hong Kong.

Dartmouth is the richer for a new cottage hospital, which has just been erected at a cost of £1,200, on the South Embankment, and was formally opened on August 1st by Mrs. Mallock, the wife of the member for the Torquay Division.

The expected "second crop" of small-pox cases has now had time to declare itself, but so far very few have been the fresh victims, and it may reasonably be hoped that the outbreak has been effectually overcome. No further deaths are reported from the hospital ships. Some 190 cases still remain under treatment.

The Asylums Committee have appointed Miss Sinclair, a fully-qualified medical woman, to the post at Claybury just vacated by Miss Annette Benson, who we mentioned last week had been elected first physician to the Kama Hospital, Bombay. Miss Sinclair has recently been attached to the staff of a fever hospital.

A local contemporary has recently called attention to the fact that certain radical improvements in the sanitary condition of the Devon and Exeter Hospital are urgently needed. If the drains are in as bad a state as they are described to be, it is certainly time that prompt and efficient steps should be taken to put matters right, at whatever cost, and no doubt the committee will see the wisdom of at once suggesting and carrying out some scheme which shall be sufficiently comprehensive to place this institution beyond the reach of criticism.

The arrangements for the Sanitary Congress which is to be held in Liverpool are now nearly complete, and it is hoped, and expected, that it will be one of the most largely attended meetings yet held by the Sanitary Institute.

The Mayor of Cork has presented to Lady Arnott, the Hon. Treasurer of the Eye, Ear, and Throat Hospital Fund a handsome pair of Sevres vases, to be offered as a prize at the forthcoming Donnybrook Fair, which is being organised in aid of the above Fund.

The International Congress of Societies for the the Prevention of Cruelty to Animals was opened at Berne last Monday. The first question of importance discussed was that of bull fights in France, and it was announced by the French delegates that a petition against the practice had been placed before the Chamber of Deputies.

The Medical Superintendent of Rainhill Asylum near Liverpool, Dr. Wiglesworth, was seriously wounded last week while going his rounds in the men's ward by a patient, who suddenly sprang upon him and stabbed him in the neck with a large wall hook which the madman had sharpened and concealed up his sleeve, severing an artery. The wound was a severe one, but Dr. Wiglesworth is now on the fair road to recovery.

As the result of a recent garden fête at Buccleuch House, Richmond, of which the expenses were entirely defrayed by Sir Whittaker Ellis, the sum of £305 13s. has been made over to the treasurer of the Royal Cambridge Asylum for Soldiers' Widows, Kingston-on-Thames. The Duke of Cambridge is President, and the Duchess of Teck, Lady President, of the Asylum. The Fishmongers' Company and the Drapers' Company contributed respectively fifty and ten guineas to this amount.

For fourteen years there has been in existence at Margate a very excellent institution, the "Clergy Seaside Rest," which does a truly good deed in enabling many inadequately-paid, over-worked, and weary parsons to enjoy the holiday and change of air and scene which is so essential for all busy toilers. There are few men, save doctors, who more badly need such a change, and few, too, who are often less able to afford it. Just at this time of year, when everyone who can has had, or is having, their yearly holiday, the committee do well to ask for pecuniary help, and we hope they may meet with due response. That the "Marine Parsonage" is appreciated may be gathered from the fact that since its opening in 1880 1,117 clergymen and their wives have availed themselves of its benefits. The apartments are quite free, a charge of 16s. per week is made for board, and 5s. for incidental expenses. Cheap railway tickets are issued to and from London at 2s. 6d. each journey, and gratuitous medical attendance is provided when needed. Only a few can be received at once, and a larger and better building, nearer the sea, is urgently needed. It is for this purpose that money is so much wanted. Contributions may be sent to the London Corresponding Secretary, the Rev. H. Jona, "Friend of the Clergy," 17, King William Street, Strand, W.C.

